# Silver linings of the Covid-19 pandemic… for some! Comparing Experiences and Social demographic characteristics of autistic and non-autistic children with SEND in England

**DOI:** 10.1007/s10803-022-05628-6

**Published:** 2022-08-09

**Authors:** Susana Castro-Kemp, Arif Mahmud ORCID

**Affiliations:** 1 UCL Institute of Education, London, United Kingdom; 2 Psychology and Human Development, 26 Woburn Square, WC1H 0AA London, United Kingdom; 3 School of Education, University of Roehampton, London, United Kingdom

**Keywords:** Covid-19, Autism, Children, Pandemic, Parents

## Abstract

Several studies on the impact of Covid-19 on children’s wellbeing have been published, including for those with Special Educational Needs and Disabilities. However, limited evidence is available on who these children may be, their socioeconomic background, age, gender or type of school attended. This study examines the role of socio-demographic characteristics on the experiences of Autistic Children, compared to non-Autistic children, to assess the detrimental impact of the pandemic, but also potential silver linings. Primary-school aged Autistic children were more likely to mention a silver lining (for mental health), as well as younger non-Autistic children from more affluent backgrounds. Similar effects were observed for older non-Autistic boys with special needs attending mainstream settings (regarding physical health).

The past two years have been very prolific in research on the impact of the Covid-19 pandemic on the lives of children and young people. Some of this research has focused specifically on the effects of the pandemic for children with Special Educational Needs and Disabilities (SEND) and their families, and some studies have provided evidence specifically for Autistic children and young people. Amongst this growing body of research, it is widely recognised that the pandemic has had mostly a negative effect on the health and education of children with SEND (particularly Autistic children and young people) and of their families. For example, in a quantitative survey of Serbian parents, concerns were reported over access to services, feelings of hopelessness and discrimination and challenges in dealing with their children’s difficult behaviour (Stankovic et al., 2021). In Saudi Arabia, the pandemic affected parental wellbeing negatively, including enhanced mental health issues and feelings of worthlessness (Althiabi, 2021), but this was dependent on severity of need and on whether symptoms were seen to have become enhanced at home (Alhuzimi, 2021). In England, a large longitudinal quantitative study with parents of Autistic children shows that these children were more negatively impacted by lockdowns, than other children with other forms of SEND, and also that this trend subsisted over time, even when schools reopened (Toseeb and Asbury, 2021). This aligns with findings from a study in China focusing on parental mental health, where parents of autistic children were more likely to have mental health problems compared to parents whose children had an intellectual disability or a visual or hearing impairment (Chen et al., 2020; Wang et al., 2021). In the United States of America (USA), parents of younger autistic children also reported higher levels of stress (Manning et al., 2021). In a large survey of parents of Autistic children who received support by a mental health unit in the USA, 59% of children were said to experience a worsening of their pre-pandemic psychiatric diagnoses and/or the development of new psychiatric symptoms during the pandemic (Vasa et al., 2021). Similarly, another large-scale survey in the USA has found that parents of Autistic children were disproportionately affected by the pandemic and its lockdowns, namely due to higher levels of overall psychological distress, hyperarousal, or feelings of panic when thinking about Covid-19 (Kalb et al., 2021). A study in Ireland also reported enhanced negative symptoms in Autistic children during the pandemic when compared to pre-pandemic times (Franz & Kelly, 2021). Negative symptoms experienced by autistic children included decreased quality of sleep, anxiety and night terrors (Bruni et al., 2021).

Less widely recognised are the findings that the pandemic and the national lockdowns may have been a silver lining for a significant minority of children and young people with SEND. A qualitative study on the experiences of parents of autistic children in Israel showed a number of fears and struggles, especially with some services not being available for some time, but also some unexpected positive outcomes, or silver linings; for example, some children improved their eating habits and routine management while at home. Similarly, for some parents who could afford the time to make the period worthwhile and were able to invest in their children’s improved routines at home, the lockdown period was rewarding and an experience of growth which fostered more independence for the children (Tokatly Latzer et al., 2021). In a qualitative follow-up of the study by Toseeb and Asbury (2021), the authors found that for a significant minority of Autistic children, the removal of the demand to attend school was a driver for wellbeing, especially when compared to children with other forms of SEND (Asbury and Toseeb, 2022). Referring specifically to physical activity for Autistic children, a qualitative study with Turkish parents showed that the pandemic and national lockdowns may have had the potential to improve physical activity at home, although certain barriers needed to be overcome (e.g. infrastructure, parental availability and lack of information), thus suggesting that in the post-pandemic world, parents could be better prepared to support physical activity of their children, provided the right facilitators are put in place (Esentürk, 2020). In Italy, Di Renzo et al., (2020) found that despite the undeniable negative impact that lockdown can have on the psychosocial well-being of children, when support was provided, this helped families to reframe the meaning of behavioural changes in their children and to understand their adaptive functionality. Hill and colleagues (2021) report that parents of autistic children found ways to provide positive home-learning environments which could be used and/or transferred back into school environments, for example, working to align the mismatch between school environments and the children’s language and functioning features. Schools’ inflexibility in adapting to individual features, over time, may be explaining why some children functioned better at home during lockdown. Asbury et al. (2020) conducted a survey with parents and reported that for a substantial minority of parents Covid-19 has had little impact on mental health in their family or has even led to improvements. Positive emotions in relation to a period of calm and respite were expressed by parents, saying children were safer and happier at home. However, who these parents are, their demographic characteristics and background, has been left mostly underexplored. In the study by Vasa et al., (2021) in the USA, it has been shown that low family income elevated the risk of enhanced psychiatric symptoms in Autistic children during the pandemic. But wider international evidence on the role that socio-demographic variables played in the experience of the pandemic is scarce and studies suggesting an effect of socioeconomic background are mainly qualitative (e.g. Tokatly Latzer et al., 2021) and therefore, with limited generalisation potential.

The present study aims to analyse the role of socio-demographic variables on the experience of Autistic children and of their families in England, during the Covid-19 pandemic, when compared to non-Autistic children and young people that have other types of SEND. The study also aims to better understand the experience of those for whom lockdown might have been seen as a silver lining (potential positive outcome) of the pandemic.

To achieve this, the following research questions were formulated: (a) do Autistic children and their families differ from children with various other SEND in how they experienced the Covid-19 pandemic, as reported by their parents, in England? (b) what were the silver linings of lockdown identified by families? (c) what is the role of socio-demographic aspects (socio-economic level of the postcode, ethnic background, age and gender) in predicting the experience of the pandemic by autistic children and young people, those with other SEND and their families, in England? (d) What is the role of type and quality of educational setting attended in predicting the experience of the pandemic by Autistic children and young people, those with other SEND and their families, in England?

## Methods

The study adopted a survey design. An online survey focusing on parental experiences of the Covid-19 pandemic was developed and issued to parents of children and young people with various SEND, between October and December 2020. The original survey focused not only on the experiences of lockdown but also of returning to school for all children with SEND and their parents (Castro-Kemp and Mahmud, 2021). In this study, we will focus on comparing the responses of parents of Autistic children versus those of parents of children with other SEND, and in relation to the experience of lockdown only. We will also explore potential silver linings of this period for these participants. The study received ethical approval from the Ethics Committee of the host organisation.

## Sample

The sample of this study is comprised of 83 parents of children and young people with SEND, who responded to the online. Participants were recruited using social media and the existing network of professional contacts and collaborations held by the authors. It is, therefore, a convenience sample. This network included parent contacts but also charities, schools and community organisations who supported the distribution and dissemination of the survey. The survey was kept open from October 2020 until early January 2021, and all respondents were included up to this point. In England, children with SEND are those that either are in receipt of a statutory document outlining their education, health and social care needs and respective provision (EHC plans), or they are included in a SEND register because they are considered to have additional needs, but do not qualify for statutory services (designated ‘SEND support’). The most common type of need for those with EHC plans is Autism Spectrum Disorders, to use the United Kingdom Government’s nomenclature (Department for Education, 2019), and therefore it is important to analyse the experiences of this particular group of children and families. The survey includes responses from parents whose children have been included in a SEND register and/or that have an EHC plan, as reported by parents in the survey. Although this is a parent-reported status, recruitment was conducted within communities of SEND services’ users, and parents were asked specifically whether their children were (1) in receipt of a statutory Education, Health and Care plan or (2) included in the SEND register at their educational institution.

## Survey

The survey was designed to be user-friendly and brief. A free online survey tool was used for this, and a mixture of open-ended and multiple-choice questions were adopted.

The items included in the survey were: (1) demographic questions concerning child’s age, gender, ethnic background, type of special need and/or disability, type of educational setting and local authority (postcode); (2) items concerning the effects of school closures: *Did your child stay at home during the spring 2020 school closures?* (multiple choice); *Do you think that school closures had a detrimental effect on your child’s mental health? Please tell us why* (multiple choice followed by open text); *Do you think that school closures had a detrimental effect on your child’s physical health?* (multiple choice); *Do you think that school closures had a detrimental effect on YOUR physical and/or mental health (please tell us why)* (multiple choice followed by open text); and (3) items on returning to school: *Now that schools have reopened, is your child back at school?* (multiple choice); *How pleased are you with the measures put in place by your child’s school to ensure Covid safety? Please tell us why* (multiple choice followed by open text); *Do you think that returning to school has had a positive impact on your child’s mental health? Please tell us why* (multiple choice followed by open text); *Do you think that returning to school has had a positive impact on your child’s physical health?* (multiple choice) and *How do YOU feel about your child returning to school?* (open text).

The survey was piloted with two parents who suggested minor changes of wording of questions and options available for multiple choice items. In this study we will focus on the responses obtained under Sect. 2) above – items concerning the effects of school closures, for Autistic *versus* non-Autistic children with SEND.

### Data Analysis

For statistical analysis, the Statistical Package for Social Sciences Software (SPSS) was used. Descriptive statistics were run for all variables under study. Additionally, to examine the predictive role of socio-demographic variables on the experience of the pandemic, series of logistic regression models were run (as all variables are categorical) considering type of need, ethnic background, socio-economic level, age, gender and type of setting attended, as predictors of effects on children’s mental health, children’s physical health and parents’ physical and mental health, as reported by the parents.

For comparison of the experience of Autistic children to the experience of children with other SEND, the item on type of SEND was recoded into a dichotomic variable (Autistic and other). Socio-economic level is given by the Index of Deprivation Affecting Children (IDACI; Smith et al., 2015), which is currently considered the best estimator of socio-economic background in England, where parental income is not available (e.g. Norbury et al., 2021; Castro-Kemp et al., 2020). Data for IDACI is available for each England borough on publicly accessible datasets on the web. This data was then recoded into three categories of ‘most affluent’, ‘mid-range IDACI’ and ‘most deprived’. Children’s age was recoded into three groups, reflecting the key-stage levels of the English Education system: preschool and key-stage 1 (3 to 7 year olds), key stage 2 (8 to 11 year olds) and key stage 3 (over 12 year olds).

Thematic analysis was used to examine the qualitative responses to the survey, by coding meaning units within participants’ responses into thematic categories, and by comparing resulting themes in the responses of Autistic children’s parents *versus* responses of parents of children with other types of SEND. This involved an iterative process of coding and categorisation, ending in a set of themes that expressed the main views put forward by participants in these items (Creswell, 2012; Guest et al., 2014). Given that the focus was on analysing a fairly small number of statements (only those for whom the pandemic did not have a detrimental effect on children’s and/or parent mental and physical health), two researchers conducted the analysis jointly, in a synchronous process of consensus. Coding was performed on a digitally shared spreadsheet. The frequencies of meaning units within themes found are reported in the results section. To enhance the trustworthiness of the coding process, the final coded spreadsheet was shared with the two non-participant parents who piloted the survey, for re-evaluating coding categories against statements provided. The involvement of members of the community of stakeholders was actively sought in the production of this paper, namely by supporting data collection and by piloting the survey issued and contributing to the analysis. These were parents of Autistic children and young people and of non-Autistic children and young people with special educational needs and disabilities. The idea for the study emerged from informal contacts and discussions with these parents. Some of them piloted the survey developed and suggested changes to wording of items. Individual parents and parent-led charities and networks supported the recruitment of participants through survey dissemination.

## Results

Table 1 summarises the sample characteristics in this study. The sample includes responses from parents of 45 Autistic children and young people and 38 non-Autistic children with various types of SEND. The sample is not equally distributed in terms of ethnicity, with significantly more non-white Autistic children (39% *versus* 14% respectively [χ^2^(1) = 5.452, *p* < .05]). Equal distribution was seen for all other variables, including age [χ^2^(2) = 0.030, *p* = .985], socioeconomic level given by the IDACI categories recoded [χ^2^(2) = 3.908, *p* = .142], gender [χ^2^(1) = 2.959, *p* = .085] and type of setting attended [χ^2^(2) = 2.223, *p* = .329].

**Table 1:** Sample Characteristics

	Type of Need				
	Autism *n* = 45	Physical and Sensory needs and Disabilities *n* = 7	Genetic Syndromes *n = 9*	Socio-emotional and behavioural issues and learning disabilities *n = 22*	Total
Ethnicity					
Any white	25	4	8	17	54
Black African and Black Caribbean	2	0	0	3	3
Any Asian	12	1	1	2	16
Any mixed	2	0	0	0	2
Age					
3 to 7	10	1	2	5	18
8 to 11	17	2	3	10	32
12 and above	18	4	4	7	33
Gender identified with					
Female	12	4	5	14	29
Male	33	3	4	8	54
Type of Educational Setting Attended					
Mainstream	22	2	4	12	40
Specialised	15	5	3	9	32
Specialised unit in mainstream	8	0	2	1	11
IDACI					
Most deprived	11	3	3	11	28
Mid-range	12	1	3	3	19
Most affluent	21	2	3	8	34

### Difference between autistic and non-autistic children in relation to the impact of lockdown

A series of binary logistic regression models were performed, where perceived children’s mental health, perceived children’s physical health and parents’ reported physical and mental health were inputted as dependent variables; gender, age, IDACI, type of setting attended, and type of need were inputted as categorical predictors. Due to low numbers of certain categories and the non-equal distribution of ethnicity, this variable was excluded from the analysis.

The borderline majority of participants (54.2%) reported that lockdown had a detrimental impact on their child’s mental health and physical health (50.6%); a large majority (62.7%) reported a detrimental impact on their own physical and mental health. No statistically significant differences were found between Autistic children and those with other types of SEND in reported children’s mental health [χ^2^(1) = 2.235, *p* = .135], children’s physical health [χ^2^(1) = 0.115, *p* = .734], or that of their parents [χ^2^(1) = 0.295, *p* = .587]. Table 2 summarises the frequency of responses to these items and the regression coefficients obtained.

**Table 2:** Regression Coefficients for effect of type of need (Autistic versus other types of need) on experience of the pandemic and lockdown

				Regression Coefficients					
		Yes	No	**B**	**SE**	**Wald**	**df**	**Sig.**	**Exp(B)**
Do you think that school closures have had a detrimental on your child’s mental health?	Autistic (indicator)	*n* = 21	n = 24	-	-	-	-	-	-
	Other SEND	*n* = 24	*n* = 14	0.673	0.450	2.235	1	0.135	1.959
Do you think that school closures have had a detrimental on your child’s physical health?	Autistic (indicator)	*n* = 22	*n* = 23	-	-	-	-	-	-
	Other SEND	*n* = 20	n = 18	0.150	0.441	0.115	1	0.734	1.162
Do you think that school closures have had a detrimental effect on your physical and/or mental health?	Autistic (indicator)	*n* = 27	*n* = 18	-	-	-	-	-	-
	Other SEND	*n* = 25	*n =* 13	0.248	0.458	0.295	1	0.587	1.282

### Reasons for potential silver linings of lockdown

The items above were followed by open-ended questions, to obtain richer and more in-depth information on the reasons behind the respondents’ ratings. Responses to these open-ended questions were analysed through thematic content analysis and themes were categorised per type of need: Autistic *versus* non-Autistic children with SEND, with a focus on those for whom the pandemic did *not* have a detrimental effect and may have had potential silver linings. Eighty-six meaning units were coded jointly by the two researchers, with fifty-three expressing a potential silver lining of the pandemic.

Amongst Autistic children and young people and those who reported no detrimental effects of the pandemic on the children’s mental health (n = 21), the main theme found was *reduced stress and anxiety* (8 meaning units). For example:

*Good for her because it removed the stress of getting to school in the mornings* (participant 9, attending a mainstream setting).

*I think my child’s nervous system re-set thanks to lockdown. Her needs weren’t met in her school and she had high level of anxiety. She was happier and calmer during lockdown because of reduced demand and expectations and reduced sensory overstimulation* (participant 20, attending a mainstream setting).

*My son loved being at home, he absolutely hated school. He would struggle going in of a morning. Now he appreciates it much more and is happier to go because he had such a long break from it* (participant 72, attending a mainstream setting).

*He has found secondary school quite stressful and not having the travel and in-school interaction with large numbers of other students has made that easier for him* (participant 83, attending a specialised unit in a mainstream setting).

The second most frequent theme amongst this sub-group of participants was *family support and safety* (7 meaning units). For example:

*Home support kept the experience supportive overall* (participant 10, attending a specialised unit in a mainstream setting)

*He felt safe and secure at home* (participant 15, attending a mainstream setting)

*She had nuclear and extended family to support her* (participant 26, attending a mainstream setting)

*He has good religion and family* (participant 74, attending a mainstream setting)

Other themes found in this set of responses were *Understanding the Situation* (2 meaning units) and *achievement and learning* (2 meaning units), for example, respectively:

*He was able to understand that he had to stay home during the school closure* (participant 40, attending a specialised unit in a mainstream setting)

*Achieved more at home than at school* (participant 1, attending a specialised setting)

Within the sub-sample of non-Autistic children and young people with SEND who reportedly did *not* suffer a negative impact on their mental health as a result of lockdown (n = 14), two of the same themes were found to those observed in the responses of Autistic children’s parents, namely *Reduced stress and anxiety* (5 meaning units) and *family support and safety* (3 meaning units). For example, respectively:

*Because she was more relaxed and the stresses and demands of school were removed. Also, she didn’t have to take her GCSE exams which was a major stressor removed* (participant 46, attending a mainstream setting).

*No because she always plays around her brothers* (participant 41, attending a specialised setting).

In addition to those themes, *School support* (2 meaning units) was also identified as a theme in this group:

*He coped well learning at home. We had contact with the school via his LSA. And he was able to Zoom and WhatsApp his friends* (participant 8, attending a mainstream setting).

*The support we received from school* (participant 58, attending a specialised setting).

Reasons put forward by parents of Autistic children and young people for how they felt the impact of the pandemic and lockdown on their own mental and physical health were no different from those put forward by parents of non-Autistic/other SEND children. Amongst those who reported no detrimental impact of lockdown (n = 31), the most frequent theme observed in their qualitative responses was *family support and safety* (10 meaning units). For example:

*It gave me and our family a chance to connect and slow down* (participant 35).

*Because I have God and family around me* (participant 75).

Participant parents also mentioned they had good *coping mechanisms* (7 meaning units):

*Had created a schedule of activities at home. Also had access to the garden to enjoy time outside* (participant 2)

*I have coping mechanisms to cope when facing challenging situations* (participant 32).

Other themes found were *physical exercise* (5 meaning units), and *a happier child* (4 meaning units). For example, respectively:

*We all were exercising regularly and knew what’s best to beat this virus. So we were OK with the school closure* (participant 5).

*Child happier and sleeping all night made for a happy house* (participant 55).

It is worth noting that not all participants who reported no detrimental impact of lockdown have provided a qualitative answer to justify this and some have provided answers that did not address the question posed. These were coded with ‘n/a – non applicable’ (8 units) and invalidated for the purpose of thematic analysis.

*The role of socio-economic level of the postcode, age, gender and type of educational setting attended in predicting the experience of the pandemic by autistic children and young people, those with other types of SEND and their families, in England*.

Table 3 summarises the results obtained in a series of logistic regression models run for the sub-sample of Autistic children and young people (*n* = 45). Amongst parents of Autistic children and young people, those with primary school-aged children (8 to 11 year olds) were more likely to report no detrimental effects of the pandemic on the children's mental health, compared to those with younger (3 to 7 year olds) and older children (12 plus); those with older boys (over 12 year olds) attending mainstream settings were more likely to report no detrimental effects on their children’s physical health, compared with those with younger children and girls. No effects of the demographic variables under study were found for parental experience.

**Table 3:** Regression Coefficients for Age, Gender, IDACI and Type of Setting Attended for Autistic CHILDREN AND YOUNG PEOPLE

		Do you think that school closures have had a detrimental on your child’s mental health?						Do you think that school closures have had a detrimental on your child’s physical health?						Do you think that school closures have had a detrimental effect on your physical and/or mental health?					
		B	SE	Wald	df	Sig.	Exp(B)	B	SE	Wald	df	Sig.	Exp(B)	B	SE	Wald	df	Sig.	Exp(B)
**Age**																			
> 12 (indicator)	-		-	4.744	2	0.093	-	-	-	6.270	2	0.044*	-	-	-	4.417	2	0.110	-
8–11	2.925		1.382	4.483	1	0.034*	18.638	2.285	1.454	2.471	1	0.116	9.828	0.535	0.977	0.300	1	0.584	1.708
3–7	0.188		0.948	0.039	1	0.843	1.206	-2.150	1.394	2.379	1	0.123	0.116	-1.464	0.924	2.510	1	0.113	0.231
**Gender**																			
Girls (indicator)	-		-	-	-	-	-	-	-	-	-	-	-	-	-	-	-	-	-
Boys	0.848		1.063	0.637	1	0.425	2.336	-3.640	1.708	4.542	1	0.033*	0.026	0.726	0.938	0.600	1	0.439	2.068
**IDACI**																			
Most Deprived (indicator)	-		-	0.464	2	0.793	-	-	-	5.624	2	0.060	-	-	-	1.257	2	0.533	-
Mid-range	-1.577		0.935	2.845	1	0.092	0.207	0.036	1.032	0.001	1	0.972	1.037	− 0.166	0.910	0.033	1	0.855	0.847
Most Affluent	-1.794		0.997	3.242	1	0.072	0.166	0.501	0.987	0.258	1	0.612	1.651	− 0.980	0.876	1.252	1	0.263	0.375
**Type of Setting**																			
Unit in mainstream (indicator)	-		-	0.464	2	0.793	-	1.338	1.449	0.853	1	0.356	3.813	-	-	0.685	2	0.710	-
Special School	− 0.628		1.190	0.278	1	0.598	0.534	-2.335	1.407	2.754	1	0.097	0.097	− 0.866	1.084	0.639	1	0.424	0.420
Mainstream	0.068		1.111	0.004	1	0.951	1.070	-3.640	1.708	4.542	1	0.033*	0.026	− 0.208	1.015	0.042	1	0.837	0.812

*Note. Significant at *p* <.05 on probability of responding ‘no’

Table 4 summarises the results obtained from a series of logistic regression models run for the sub-sample of children and young people with other types of SEND. Here, no effects were found on children’s mental health or parents’ physical and mental health. Regarding children’s physical health, parents of younger children (3 to 7 year olds) and from more affluent backgrounds were more likely to report no detrimental effects from lockdown, when compared to those with older children and from more deprived or mid-range IDACI postcodes. No effects were found for the parents’ own experience.

**Table 4:** Regression Coefficients for Age, Gender, IDACI and Type of Setting Attended for CHILDREN AND YOUNG PEOPLEWith Various Types of Need Other Than Autism

	Do you think that school closures have had a detrimental on your child’s mental health?						Do you think that school closures have had a detrimental on your child’s physical health?							Do you think that school closures have had a detrimental effect on your physical and/or mental health?						
	B	SE	Wald	df	Sig.	Exp(B)	B	SE		Wald	df	Sig.	Exp(B)	B		SE	Wald	df	Sig.	Exp(B)
**Age**																				
> 12 (indicator)	-	-	4.540	2	0.103	-	-	-		5.935	2	0.051	-	-		-	0.060	2	0.970	-
8–11	0.330	1.078	0.094	1	0.0760	1.391	-1.936	1.284		2.273	1	0.132	0.144	0.031		1.188	0.001	1	0.979	1.032
3–7	-2.140	1.124	3.628	1	0.057	0.118	-3.083	1.265		5.935	1	0.015*	0.046	0.225		1.019	0.049	1	0.825	1.253
**Gender**																				
Girls (indicator)	-	-	-	-	-	-	-	-		-	-	-	-	-		-	-	-	-	-
Boys	0.459	0.953	0.232	1	0.630	1.582	− 0.983	0.999		0.969	1	0.325	0.374	0.409		0.949	0.186	1	0.666	1.506
**IDACI**																				
Most Deprived (indicator)	-	-	2.848	2	0.241	-	-	-	4.061		2	0.131	-	-	-		0.276	2	0.871	-
Mid-range	-1.864	1.106	2.842	1	0.092	0.155	-8.95	0.959	0.872		1	0.351	0.408	0.098	0.888		0.012	1	0.912	1.103
Most Affluent	-1.094	1.163	0.885	1	0.347	0.335	-2.896	1.438	4.056		1	0.044*	0.055	-5.99	1.386		0.187	1	0.665	0.549-
**Type of Setting**																				
Unit in mainstream (indicator)	-	-	0.718	2	0.698	-	-	-	1.979		2	0.372	-	-	-		0.110	2	0.947	-
Special School	1.389	1.655	0.704	1	0.402	4.009	1.244	2.063	0.363		1	0.547	3.469	-22.067	23013.280		0.000	1	0.999	0.000
Mainstream	1.046	1.625	0.414	1	0.520	2.846	− 0.010	2.000	0.000		1	0.996	0.990	-22.367	3013.280		0.000	1	0.999	0.000

*Note. Significant at *p* <.05 on probability of responding ‘no’

## Discussion

This study aimed to analyse the role of socio-demographic variables on the experience of Autistic children and their families in England, during the Covid-19 pandemic, compared to non-Autistic children and young people with other types of SEND. Moreover, it aimed to better understand the experience of those for whom lockdown might have been seen as a silver lining of the pandemic.

Results indicate no difference between Autistic children and non-Autistic children with SEND in relation to how they experienced the pandemic and the first national lockdown, in terms of their mental and physical health, as reported by their parents. Equally, there is no difference between the parents of the two groups of children in how they experienced the pandemic themselves. It is worth noting though, that similar to what has been indicated in previous studies (e.g. Neece et al., 2020), a large proportion of parents reported that lockdown did not have a detrimental impact on their children’s health. The examination of their qualitative responses provides greater insight into this experience and supports the idea previously put forward that for many, lockdown may have provided a silver lining (e.g. Chawla et al., 2020). Many parents reported reduced stress and anxiety expressed by their children, a happier and calmer atmosphere overall, and some explicitly said school was not meeting their child’s needs, and therefore, at home, they were happier and more functional. Responses and themes encountered in these responses were mostly similar between the two groups of children (Autistic *versus* non-Autistic with SEND), although non-Autistic children’s parents commented more often on the importance of school support, as a positive aspect for those who did not feel a negative impact.

We looked at who these participants were, considering a number of socio-demographic variables (socioeconomic level of the postcode, age and gender of the child and type of setting attended). We found that amongst parents of Autistic children and young people, those with primary school-aged children (8 to 11 year olds) were more likely to report a silver lining of lockdown on their children’s mental health, or at least no detrimental impact, compared to those with younger (3 to 7 year olds) and older children (over 12 years old). It is possible that this age group represents a period where supports and provision have been put in place and transferred to the home environment, routines have been established, but the added pressures of adolescence-related challenges are not present yet. On the other hand, the children are old enough to follow those established routines at home with a certain level of independence, compared to younger ones, who may have a recent diagnosis. However, this was not observed in non-Autistic children with other SEND. This may be related to a wide variety of functional needs represented in this group, some of which may be medical needs that make the lack of access to services a more significant issue. In this group, older boys attending mainstream settings were more likely to report no detrimental effects on their physical health, compared to younger children and girls. This is potentially due to the fact that older boys may typically develop more consistent outdoor/exercise habits than younger boys and girls, especially if they attend mainstream settings, as they are more likely to present a more adaptable functioning profile, than those attending specialised settings. This is true given the United Kingdom Governments’ statistics suggesting that children with statutory support (and therefore requiring more support) are more likely to attend specialised settings (UK Government, 2020).

More prominently, younger non-Autistic children with SEND (including children with a range of physical, genetic and sensory needs) from more affluent backgrounds are significantly more likely to report no detrimental impact of lockdown. This is suggestive of the difficulties faced by those in deprived households, with more challenging access to services and childcare; in a recent paper by the authors where parental experiences of lockdown were explored, ‘Financial worries’ was one of the frequent themes found in the responses from this group of children’s parents (Author, author, 2021).

This study provides insight into aspects that may have influenced the experience of the pandemic and lockdown for Autistic and non-Autistic children with SEND, but it is suggestive that the lived experiences of these two groups are relatively similar, as reported by parents. This contradicts previous studies that have shown a more challenging experience had by Autistic children (e.g. Chen et al., 2020; Wang et al., 2021). However, this may be due to the relatively small sample adopted between groups. It is also well known that parents from lower socio-economic backgrounds are less likely to engage in research, especially when conducted online (Thorell et al., 2021), and therefore a larger sample would perhaps have captured a larger effect between groups. Another possible limitation of this study is that parental report may not reflect the objective levels of child stress and wellbeing, but rather parental experiences only. This may also influence the objective levels of stress experienced by children and young people themselves, for example, Corbett et al., (2021), studied stress level in Autistic youth and their parents and observed that adult stress was the primary predictor of parent perception of child stress as well as child self-reported stress. Lastly, it is important to note that in England there is no registered and/or official documentation on children’s functioning, regardless of whether they have statutory or non-statutory support. Therefore, it is difficult to ascertain whether children in the Autistic group have co-morbidities that makes them similar, in functioning terms, to many children in the non-Autistic group, making potential differences between groups less apparent. The English policy for SEND has been widely criticised for this medicalised approach to eligibility (e.g. Castro & Palikara 2016). We do know, however, that because children with statutory support are generally those with more explicit functioning difficulties, and the most common type of need in this group is ASD, it would be expected to see a clearer difference between Autistic and non-Autistic. Nevertheless, in the present study, diagnosis itself, in the presence of additional needs, does not seem to relate to a different impact on children’s mental and physical health and that of their parents. It is the role of other variables such as age, type of setting attended and socio-economic level that may explain differences observed, which has been scarcely documented to date. This is an important finding, as it demonstrates that children are not being provided with equal opportunities and supports to back their learning and development throughout the pandemic, as clearly expressed by some of the respondents. It may also be revealing systemic problems in the educational, health and social care structures that stem from pre-pandemic times. The fact that so many parents refer to lockdown as a silver lining, a period where children were calmer, less anxious and agitated, and the family had more quality time together, suggests that schools and services were not previously meeting everyone’s needs effectively. The mere accessibility to services does not ensure all children are experiencing them positively and the pandemic associated lockdowns may have revealed those idiosyncrasies. Moreover, for some of those in more affluent background, being outside school was better. This is a strong indication for a need to reform organisational structures so that public services meet everyone’s needs. Future research should address this issue in larger sample and with additional indicators of socio-economic status rather than IDACI alone; although used often in England as a reliable indicator, it is based on average rankings of postcodes, rather than objectively measuring each family’s socio-economic standing, which could potentially be seen as a limitation. Despite this, schools, services and provision teams should adjust to the new reality of the pandemic and its unpredictable nature to ensure that the individual functional needs of children are being met adequately and fairly, across all strands of society. Narzisi (2020) reflected on ‘creative adjustments’ that ought to be made in provision of services for Autistic children and their families, to be better prepared and ‘closer’ to their needs in contexts of crisis; the author provides tips to providers, including weekly online consultations with parents, online therapy for high-functioning children, shared videos and internet sessions with parents and serious games, amongst other creative solutions, which perhaps would not be so promptly considered before the pandemic. Schools and services must take lessons learned from the (ongoing) pandemic to be better prepared for a crisis, and to be able to reach all children and families equally. Chawla and colleagues (2020) explain that lockdown provided children with ‘free time’ to spend on developing other creative skills, which otherwise they would not have the opportunity to engage in. This is not meant to downplay the very harmful and documented impacts of the pandemic, but to highlight the need to learn lessons from this life-changing event and re-create schools as places and institutions that serve all, flexibly and in an adaptive manner. The present study adds to the available evidence that the pandemic has highlighted the need to dismantle systemic inequities within our educational systems (Hill et al., 2020) and best use of technology can help bridge gaps. For example, Guido-Estrada & Crawford (2020) designate telemedicine as a silver lining of the pandemic that must be embraced. Its capacity to serve large catchment areas has the enormous potential to reach those in most deprived and/or rural areas, enhancing equality of opportunity with a potentially cost saving service. The future should entail experimenting with new solutions and continuously assess individual experience in an effort to become closer and closer to a ‘level playing field’.

Lastly, it would be important to see similar studies conducted in other countries, to fully appreciate the extent of social inequality’s role on learned lessons from the pandemic, internationally.

## Conclusions

This study aimed to analyse the role of socio-demographic variables on the experience of Autistic children and their families in England, during the Covid-19 pandemic, compared to non-Autistic children and young people with other types of SEND. It also aimed to better understand the experience of those for whom lockdown might have been seen as a silver lining of the pandemic. A large proportion of parents reported that lockdown did not have a negative impact on children, in line with existing literature (e.g. Chawla et al., 2020). Results show that although the type of need (Autistic versus non-Autistic) is not a main predictor of the children’s experiences, some effects of socio-demographic variables are seen differently in the two groups: primary-school aged (8–11 year olds) Autistic children were more likely to mention a silver lining in relation to their mental health, as well as younger non-Autistic children (3 to 7 year olds) from more affluent backgrounds. Similar effects were observed for older non-Autistic boys (12 plus) with special needs attending mainstream settings, when considering their physical health. Parents highlight mainly reduced child stress and anxiety at home, and more positive family time, when referring to lockdown as a silver lining. Financial worries and pressure of doing too many jobs were common themes amongst those who experienced detrimental effects. These results are indicative of systemic problems in the educational, health and social care structures that stem from pre-pandemic times. The large number of parents referring to lockdown as a silver lining, a period where children were calmer, less anxious or agitated, and the family had more quality time together, suggests that schools and services were not meeting everyone’s needs effectively, prior to the pandemic. Moreover, for some of those in more affluent backgrounds, being outside school was better. This is a strong indication for a need to reform organisational structures so that public services meet everyone’s needs more effectively, making used of lessons learned from the pandemic, including the important role that technology can play (Narzisi, 2020).
